# *Cassini*/VIMS observes rough surfaces on Titan’s Punga Mare in specular reflection

**DOI:** 10.1186/s13535-014-0003-4

**Published:** 2014-08-21

**Authors:** Jason W Barnes, Christophe Sotin, Jason M Soderblom, Robert H Brown, Alexander G Hayes, Mark Donelan, Sebastien Rodriguez, Stéphane Le Mouélic, Kevin H Baines, Thomas B McCord

**Affiliations:** Department of Physics, University of Idaho, Moscow, 83844-0903 Idaho USA; Jet Propulsion Laboratory, Caltech, Pasadena, 91109 California USA; Department of Earth, Atmospheric, and Planetary Sciences, Massachusetts Institute of Technology, Cambridge, 02141 MA USA; Bear Fight Institute, Winthrop, 98862 Washington USA; Lunar and Planetary Laboratory, University of Arizona, Tucson, 85721 Arizona USA; Laboratoire AIM, Université Paris Diderot/CEA Irfu/CNRS, Centre de l’orme des Mérisiers, bât. 709, Gif/Yvette Cedex, 91191 France; Laboratoire de Planétologie et Géodynamique, CNRS UMR6112, Université de Nantes, Nantes, France; Department of Astronomy, Cornell University, Ithaca, 14853 NY USA; Space Science and Engineering Center, University of Wisconsin, Madison, 53706 WI USA; University of Miami, Miami, 33149 FL USA

**Keywords:** Titan, Hydrology

## Abstract

*Cassini*/VIMS high-phase specular observations of Titan’s north pole during the T85 flyby show evidence for isolated patches of rough liquid surface within the boundaries of the sea Punga Mare. The roughness shows typical slopes of 6°±1°. These rough areas could be either wet mudflats or a wavy sea. Because of their large areal extent, patchy geographic distribution, and uniform appearance at low phase, we prefer a waves interpretation. Applying theoretical wave calculations based on Titan conditions our slope determination allows us to infer winds of 0.76±0.09 m/s and significant wave heights of $2^{+2}_{-1}$ cm at the time and locations of the observation. If correct, these would represent the first waves seen on Titan’s seas, and also the first extraterrestrial sea-surface waves in general.

## Background

Saturn’s moon Titan posesses the only known open surface liquids beyond Earth [1]. Those liquids take the form of lakes made primarily of methane, ethane, and dissolved nitrogen [2]. The bulk of the volume of Titan’s liquids occurs near the north pole [3,4], though isolated lakes have also been observed near the south pole at Ontario Lacus [5–8], possibly near the equator [9], and southern mid-latitude (Sionascaig Lacus) (Vixie G, Barnes JW, Jackson B, Wilson P: Two temperate lakes on Titan. Icarus, submitted).

The existence of extraterrestrial lacustrine environments allows for the possibility of waves. In theory, expanses of liquid acted upon by sufficient winds ought to show the formation of waves on Titan as they do on Earth. The wind velocity needed to generate such waves, along with the resultant wave frequencies, will necessarily be affected by Titan’s alien gravity, atmospheric density, and liquid viscosity/surface tension (which are in turn a function of composition and temperature). Theoretical calculations [10–12] predict that the first waves to be incited on Titan when the winds break the threshold should occur with wavelengths between 2.8 cm and 3.2 cm. Hayes et al. [12] show that these waves should be capillary-gravity waves — ones for which surface tension and gravity both contribute to the restoring force. Initial laboratory experiments with kerosene in a wind tunnel [13] showed that waves on hydrocarbons are both larger than waves on water and form at lower wind speeds, at least under Earth gravity and atmospheric conditions.

Despite concerted efforts, however, *Cassini* has thus far not detected any waves. Brown et al. [2] showed that near-infrared spectral determinations of the reflectivity of Ontario Lacus were consistent with a “smooth” surface. Wye et al. [14] used direct reflection of *Cassini*’s RADAR off Ontario Lacus to constrain the surface roughness to be less than 3 mm(!). Barnes et al. [15] used a time-resolved specular reflection across north polar Jingpo Lacus to constrain wave angles at that time to be less than 0.15°. All of these observations are consistent with Titan lakes that are as flat as a millpond at the time of the observations: entirely wave-free [12].

These nondetections notwithstanding, there is indirect evidence to support the hypothesis that waves do form on Titan’s lakes and seas. Wall et al. [16] claimed geomorphological evidence for waves, suggesting that the eastern shore of Ontario Lacus represents a beach formed by wave-deposited sediments. Lorenz et al. [17] explored possible explanations for why no waves have been seen, suggesting a seasonal effect, i.e. that the winds above Titan’s lakes and seas were too low at the time of the observations to initiate wave formation. Calculations suggest that the threshold wind speed for wave formation under Titan conditions might be between 0.4 m/s and 0.8 m/s [12,17]. General Circulation Models (GCMs) generally predict that the winds near Titan’s north pole should have been rather quiescent until late northern spring, consistent with the low-wind explanation for Titan’s lack of waves thus far [17,18].

In this paper, we report evidence for waves on Titan’s northern sea, Punga Mare. In the ‘Observation’ section we describe the *Cassini* Visual and Infrared Mapping Spectrometer (VIMS) data that we interpret to show the waves in specular reflection. We describe our model for simulating the appearance of roughness-driven specular reflections away from the specular point in the ‘Model’ section. In the ‘Analysis’ section we apply that model to the VIMS observations to derive wave properties. Then, in the ‘Discussion’ section, we consider the implications of the discovered waves, before concluding.

## Observation

**Figure 1:**
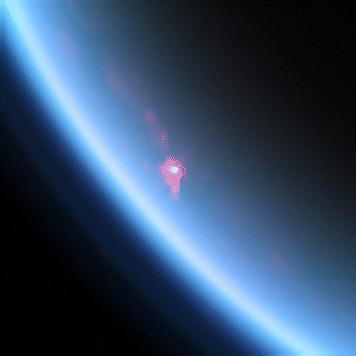
**Cube CM_1721848119_1.** *Cassini* Visual and Infrared Mapping Spectrometer (VIMS) cube CM_1721848119_1 from the T85 Titan flyby on 2012 July 24. This cube shows a bright specular reflection (sunglint) off Kivu Lacus. This interpolated color version uses 2.0 μm as blue, 2.8 μm as green, and 5.0 μm as red. The complex structure surrounding the central glint is described in Barnes et al. ([20]).

In Figure 1 we show VIMS [19] cube CM_1721848119_1, acquired during the T85 flyby on 2012 July 24. It shows a very bright specular reflection (in white in Figure 1 off a lake at 87.5°N called Kivu Lacus, which was previously used to derive an atmospheric transmission spectrum [20]). This cube was acquired from a range to Titan closer than any other specular reflection observation to date (30000 km). It is this close range that makes the reflection so bright [21].

**Figure 2:**
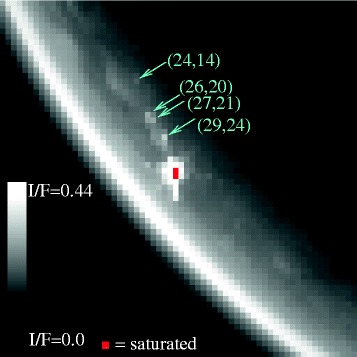
**5- μm image.** Here we show the 5- μm window of cube CM_1721848119_1, scaled from *I*/*F*=0.0 to *I*/*F*=0.44. Red indicates the saturation of pixels, which occurs at the primary specular reflection off Kivu Lacus. The arrows indicate the areas of interest for this paper, which show specular reflections on Punga Mare away from the specular point that may represent wave activity.

**Figure 3:**
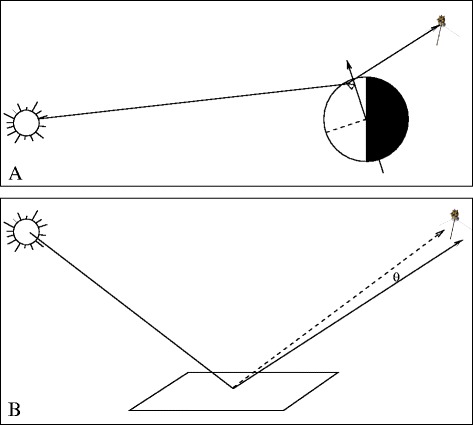
**Observation geometry.** This cartoon illustrates the geometry of the T85 VIMS observation. At top **(Panel A)** is the large-scale geometry with the Sun at left and *Cassini* at right. At the bottom **(Panel B)** is the geometry for a given pixel. Distances are not to scale in either diagram.

While the Kivu specular reflection can be seen at 2.0 μm, 2.7 μm, and 2.8 μm as well, at 5 μm it is so bright that we can see additional effects over and above the raw glint (Figure 2). The specular reflection appears extended at 5 μm because of forward-scattering of specularly-reflected sunlight from haze in the atmosphere above the lake [20]. The central specular pixel and its neighbor are saturated at 5 μm and 2.8 μm. Figure 2 also shows an unusually high (though unsaturated) signal in a few pixels that are a considerable distance away from the specular point (cyan arrows in Figure 2). We show a diagram of the geometry of the T85 Kivu observation in Figure 3.

**Figure 4:**
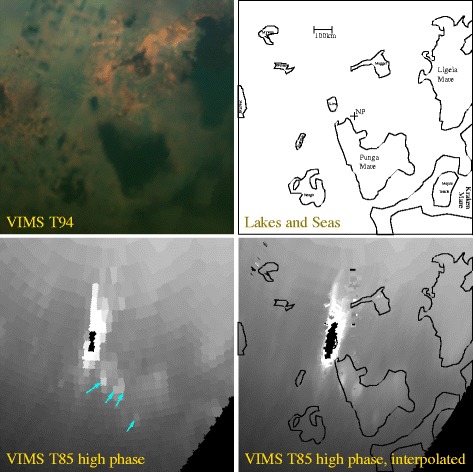
**North polar projections.** Four different orthographic projections of Titan’s north polar region. At top-left is a near-infrared color version acquired on T94 with 5 μm as red, 2.0 μm as green, and 1.3 μm as blue. At upper-right we show the outlines of various named features discussed in the text. A 5 μm version of cube CM_1721848119_1 from Figure 2 is shown Lanczos interpolated at lower-right, and as pixels in the lower-left. Features with just one name are designated ‘Lacus’. For purposes of these images, saturated pixels are assigned a highly negative *I*/*F* to avoid contamination when used in the interpolation.

**Figure 5:**
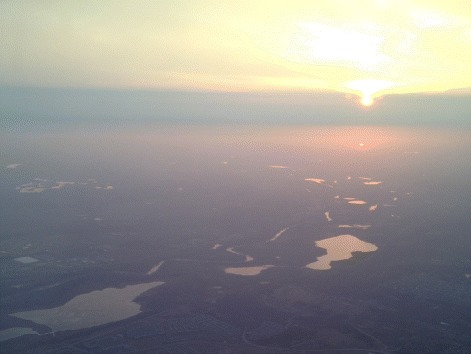
**Lakes in twilight.** Photo looking west out the left-hand window of an aircraft on approach to Minneapolis/St. Paul airport. The Sun is just setting. The dark areas seen on the ground are solid land. The bright areas are lakes that are specularly reflecting bright ambient sky illumination.

To place the off-specular-point areas of high signal into context, we show a mapped version of the T85 data for cube CM_1721848119_1 along with a more recent fine-resolution view from low emission angle that VIMS acquired on T94 (2013 September 12) in Figure 4. Of particular interest to note is that, in contrast to how Titan’s lakes and seas appear at low phase angle, in the T85 CM_1721848119_1 high phase angle (148°) observations Titan’s liquid expanses (lakes and seas) appear brighter than the surrounding terrain. This contrast inversion occurs because VIMS is seeing a specular reflection of the (somewhat) bright sky from the lake and sea surfaces [6,17]. We show a practical demonstration of this effect in Figure 5.

The bright lakes and seas do not all show the same measured *I*/*F*. There are three reasons for this. The first is that at 5 μm Titan’s atmosphere is optically thin. So if you were standing on the surface in a boat on one of these lakes you would see that the sky was brighter near the horizon than at the zenith. Because lower emission angles specularly reflect a portion of the sky nearer the zenith, those areas necessarily show a dimmer specular sky reflection. The second reason is that the efficiency of the specular reflection decreases as the emission angle decreases [21]. Finally, the T85 view encompasses the terminator, meaning that past 90° incidence angle there is no direct flux at all in the lower atmosphere.

However all three of these effects are continuous and would not lead to any liquid-filled areas differing significantly in brightness from neighboring areas in a discontinuous manner. In particular, bright specular sky reflections seen in Ligeia Mare and Kraken Mare are continuous, without spurious brighter or darker areas within the seas. Kraken does have a darker area corresponding to the island Mayda Insula. Smaller lakes Sparrow Lacus, Waikare Lacus, and Muggel Lacus each are detectably brighter than their surroundings. Neagh Lacus is beyond the terminator.

**Figure 6:**
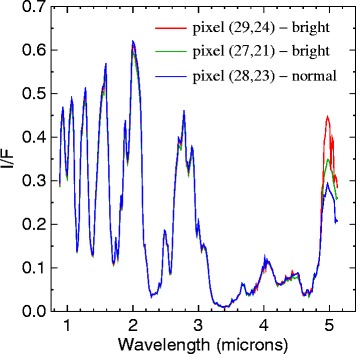
**Near-infrared spectrum.** VIMS Near-IR spectrum of three pixels from cube CM_1721848119_1. Two of the bright pixels from the cyan arrows in Figure 2 are shown in red and green, and a nearby reference pixel is in blue. The spectral signature whereby the bright areas are brighter only in the 5 μm window is consistent with their being due to specular reflection.

The areas shown with cyan arrows in Figure 2 indicate three separate areas within Punga Mare with discontinuous and anomalously bright *I*/*F*. Two of these are at the northern end of Punga, and one is in the south. To evaluate the nature of these anomalously bright pixels, we show their spectra in Figure 6. The spectral signature shows that the bright pixels are bright only within the 5 μm window. This pattern matches the expected distribution for a specular reflection viewed through Titan’s hazy atmosphere [21,22]. VIMS does not have polarization capability, so all fluxes are total light intensity.

In particular, these spots’ spectra are not consistent with an isolated patch of fog or other atmospheric aerosols above the lake — such a patch would be expected to show significant signal at wavelengths shorter than 5 μm as well [23–25]. While lakes and seas can reflect the image of background clouds, VIMS imaging in the wings of the 2 micron window show no evidence of cloud activity in the area at the time of the T85 observation. Hence while we cannot be certain that the spurious 5-micron flux derives from surface specular reflection, all of our tests are consistent with a surface specular phenomenon.

How can this flux be specular in origin, though, if the pixels themselves are not at the specular point on Titan’s surface? Calculation of the surface specular point assumes that the surface conforms to a local equipotential surface. If the surface is instead tilted, or if portions or facets of the surface are so tilted, then either it or some of its facets can achieve a specular geometry away from the nominal specular point.

**Figure 7:**
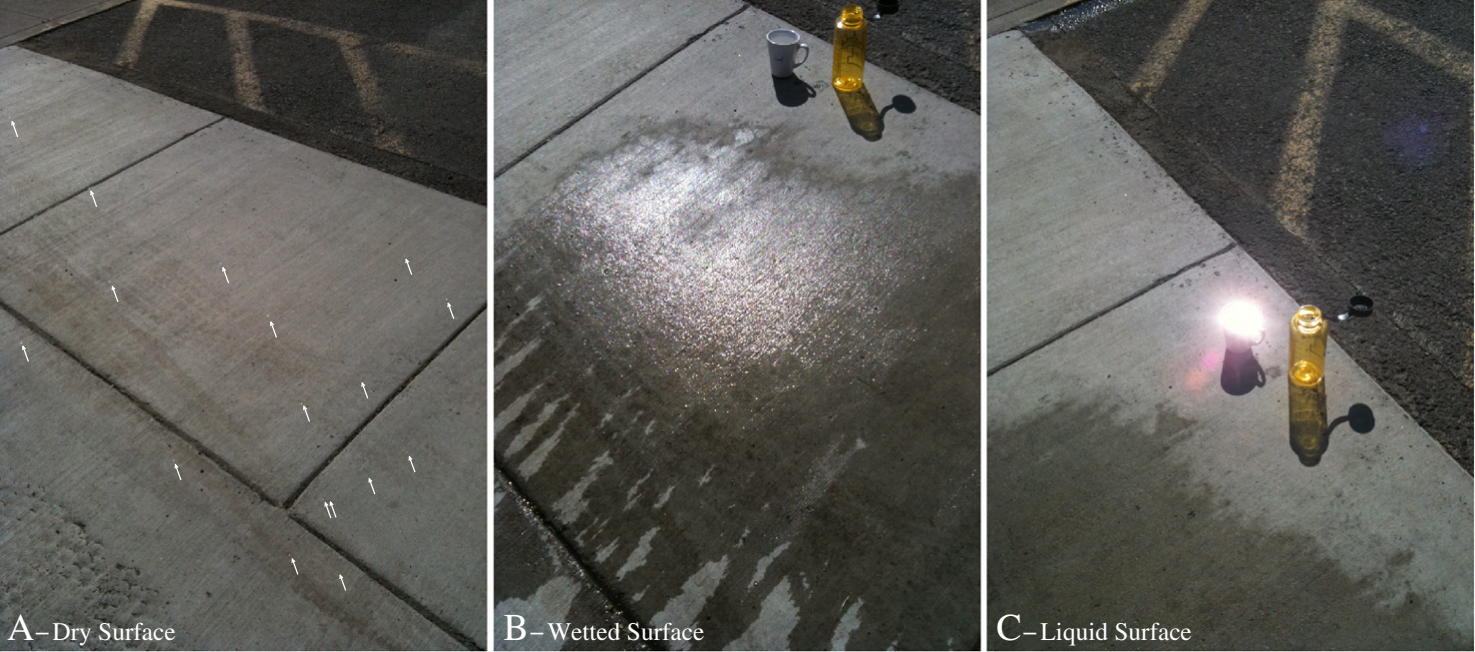
**A sidewalk experiment.** Different types of specular reflection as viewed on a sidewalk on the University of Idaho campus. **(A)** Solid surfaces can produce specular reflections if their surfaces are smooth at relevant wavelength provided that they are oriented in the correct direction to specularly reflect the Sun into the observer’s direction. In this case, each little arrow points to a bright speckle coming from a smooth piece of gravel sticking out of the concrete. **(B)** Here water has been dumped onto the surface. The water in this case follows the rough contours of the concrete, but the water’s surface tension keeps it smooth at visible wavelengths and the index of refraction allows for a strong specular reflectivity. Hence there is a specular reflection, but a broad one due to some angled facets having specular geometry even away from the central specular point. **(C)** This image shows a specular reflection from perfectly smooth water inside the first-author’s *Kepler* coffee mug. The specular signal is concentrated at a single point, and is particularly bright when compared to the weak signal from the solid surface in A.

We show an illustration of this effect in Figure 7. Figure 7C shows a tight specular reflection of the Sun from a smooth surface, while Figure 7B shows a broad specular reflection from a wetted rough surface. The specular reflection from the rough surface extends over several degrees, but is most intense when the surface is not just moist, but is actually covered by a thin layer of liquid.

We thus suggest that the bright, specular pixels within Punga Mare in Figure 2 could represent a rough, wet surface at those particular locations.

## Model

We develop a numerical model of planetary specular reflections to evaluate whether the brightened areas in Figure 2 could plausibly result from specular reflection from a rough, wet surface. We use the SPICE package [26] combined with a downhill simplex numerical minimization algorithm [27] to calculate the precise orientation for a specular facet at any given point on Titan’s surface given a specific observation geometry. For each latitude/longitude point, we vary both the angular deviation from zenith (*θ*) and azimuthal orientation (*φ*) for which the angular distance between the specular vector and the Sun direction vector is zero.

**Figure 8:**
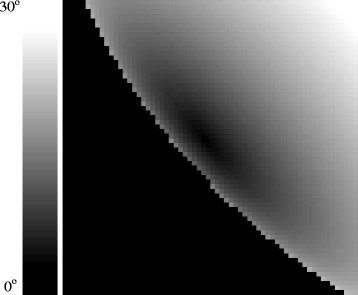
**Surface facet angular deviation.** This image, which corresponds to the pixel geometry from cube CM_1721848119_1 as shown in Figure 1, represents how far the orientation of a surface facet would have to be tilted at each location in order to achieve a specular geometry with the Sun. This value, which we call the specular facet deviation *θ*, varies across Punga Mare from 3.4° at the end nearest to Kivu Lacus to 10° at the far end.

In Figure 8 we show the result of such calculations for *θ* as applied to the specific geometry in VIMS cube CM_1721848119_1. Due to the nature of specular reflection and the close observation geometry, the angular deviations required are roughly symmetric in the azimuthal direction around Titan’s disk, but not in the radial direction. Furthermore, the radial and azimuthal directions are not symmetric with one another, either.

To then calculate the expected brightness in *I*/*F* at each point under the roughened liquid specular scenario, we calculate the fraction of randomly oriented facets that would achieve specularity with some portion of the extended solar disk. This calculation requires an assumption for the distribution of the orientation of facets within the pixel. For this work (as in [15]) we assume a two dimensional Gaussian distribution with varying widths *σ*. Although this assumption about the distribution may not provide the highest fidelity, given our modest 4 pixels we elect to leave a more realistic distribution including wind directionality to future work as data warrant.

**Figure 9:**
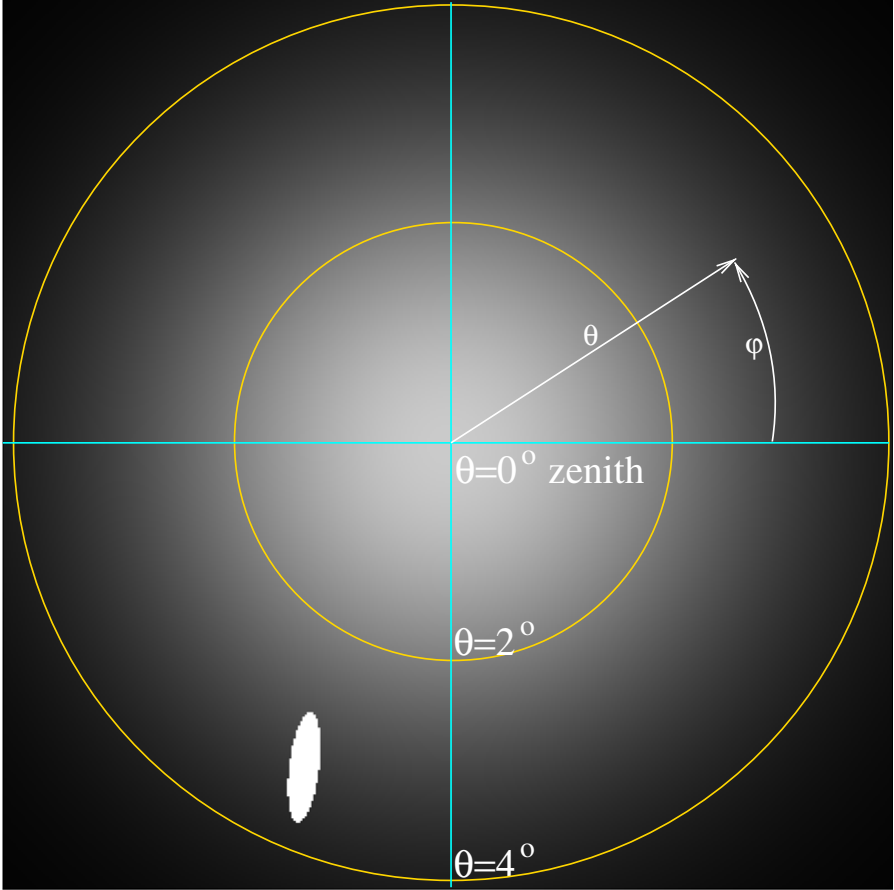
**Modeling facet directions.** Synthetic map in *θ*- *φ* space showing the relative probability of an individual surface facet facing in any particular direction for a single particular location on Titan’s surface during the T85 CM_1721848119_1 observation. This map uses *σ*=2°. The white oval represents the area over which the specular vector from the surface intersects the solar disk. For this particular figure the size of the solar disk has been exaggerated by a factor of 10 for visibility.

Figure 9 shows the result of one such calculation for a particular pixel, assuming that width of the typical facet deviation *θ* distribution is *σ*=2°. The brightness in this image represents the relative probability for a facet to be oriented in any given direction from the zenith (*θ*) and azimuthally (*φ*) with *θ*=0.0° at the center of the image. The gold circles represent lines where *θ* is an integral multiple of *σ*. The white oval in the lower-left quadrant represents those facets for which the specular direction points within the Sun’s disk (for the purposes of illustration the Sun’s angular diameter has been multiplied by 10 for visibility).

The fraction of facets *f* that should show specular reflection at each pixel is equal to

(1) $$ f(\theta,\varphi) = \int_{0}^{\pi}\int_{-\pi}^{\pi}G(\theta)\Gamma(\theta,\varphi)~2\pi\theta d\varphi~d\theta  $$

where *G* is a two-dimensional gaussian function equal to

(2) $$ G(\theta) = \frac{1}{2\pi\sigma^{2}}e^{-\frac{\theta^{2}}{\sigma^{2}}}  $$

and *Γ*(*θ*,*φ*) is a function equal to 1.0 when the facet at (*θ*,*φ*) corresponds to a specular direction inside the solar disk, and equal to 0.0 outside it.

For purposes of calculating the fraction of specular facets numerically, we break the solar disk into a 30-sided polygon with the vertices around the Sun’s limb. We then calculate the area of that 30-sided polygon in *θ*- *φ* space and multiply the area by the Gaussian distribution value corresponding to the center of the Sun. This technique is much faster than an explicit two-dimensional numerical integral. Furthermore, given that the angular diameter of the Sun as seen from Titan (0.05°) is much smaller than the typical width of the facet deviation distribution that we explore (*σ*=1−10°) the approximation is highly accurate as well.

Finally, we normalize the total integral of the Gaussian facet deviation distribution to an assumed overall *I*/*F* value. Doing so obviates the necessity of making assumptions regarding the liquid’s index of refraction and therefore of its precise composition. The final pixel value then is equal to the total solar specular flux parameter times the fraction of specular facets — this ensures that in the case where *σ*=0°, the specular *I*/*F* of the pixel at the specular point would be *I*/*F*_max_. Note that because the original and model pixel *I*/*F* values are normalized, and are not true measured fluxes, summing *I*/*F* over the affected pixels does not yield *I*/*F*_max_.

**Figure 10:**
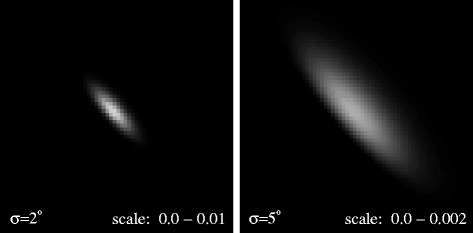
**Model specular reflections.** Synthetic images of specular flux from a uniform global ocean with Gaussian-distributed slopes of *σ*=2° (left) and *σ*=5° (right). These images match the viewing geometry of cube CM_1721848119_1 as shown in Figure 2.

We show sample results from this model in Figure 10 for *σ*=2° and *σ*=5°. Of particular note is the stretch in each synthetic image — for higher dispersion in specular deviation angle (higher *σ*) the total specular reflection becomes fuzzier, but importantly also diffuses the flux over a greater number of pixels. Hence each individual pixel shares a smaller total specular flux in the high *σ* case. Once we isolate the specular signal from the Punga Mare pixels, we can compare them to the model to infer surface roughness and evaluate the veracity of the roughened liquid model.

## Analysis

**Figure 11:**
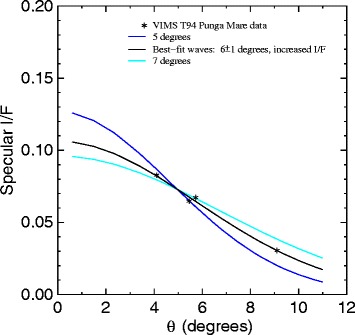
**Wave fit.** Plot of the specular flux from 4 points on Punga Mare at 5 μm from cube CM_1721848119_1 (asterisks) as a function of the specular facet deviation *θ*, along with a line representing the result of a *χ* ^2^ minimization fit with *σ*=5.8°.

To isolate the fraction of flux coming from the off-specular specular reflection of the Sun, we subtract an estimated background flux from each of our 4 pixels of interest. The backgrounds were taken from adjacent pixels within the lake but not showing Sun specular signal. For pixel (29,24), closest to Kivu Lacus, we used the average of pixels (29,23) and (29,25) (see Figure 2 for location of pixels). All pixel locations are measured from the upper-left with zero-offset arrays (i.e. the first pixel is pixel number zero). For both pixel (27,21) and (26,20) we used the average of (26,19) and (27,22). And for the pixel (24,14) at the south end of Punga Mare we used the average of pixels (23,13) and (25,15). Properly subtracted and averaged over the 16 VIMS channels within Titan’s 5 μm atmospheric window, we arrive at the data points shown as asterisks in Figure 11. We assume that each pixel represents a uniform patch all with the same wave facet distribution.

To fit these data, we drove the specular brightness model with a Levenberg-Marquardt *χ*^2^ minimization fit [27] to fit for both the maximum *I*/*F* and the facet distribution width *σ*. The best-fit values that result from that fit are *I*/*F*_max_=106±16 and *σ*=6°±1°. The best-fit line (based on an assumed approximately radial profile path away from the specular point) is shown in black in Figure 11.

It is not clear why the best-fit value for *I*/*F*_max_ is discrepant from the Barnes et al. [20] value (*I*/*F*=32.4) by a factor of 3. The main specular pixel is saturated, so the error could be a result of the Barnes et al. 2013 reconstruction of the saturated values. It could also be that the true surface facet distribution does not match the 2-D Gaussian that we have assumed or that the liquid surface of Kivu Lacus where the prime specular reflection was measured is partially covered with some kind of opaque film (pond scum). Differences in the real index of refraction caused by composition differences between Kivu Lacus and Punga Mare cannot account for the offset, as at this phase angle, the first-surface reflectance of pure methane and that of pure ethane differ by only ∼20% [21]. We note, however, that this new value of *I*/*F*_max_ is closer to the theoretical value as derived using the Soderblom et al. [21] relations of ∼70 for methane and ∼90 for ethane than that from the Kivu Lacus observation [20].

## Discussion

Our fit shows that the flux coming from the three isolated areas of Punga Mare studied is consistent with a rough liquid surface having characteristic slopes of 6°. Such surfaces could come about as a result of wetted mudflats, for instance, similar to the wet sidewalk in Figure 7B. A bright, dry playa surface could generate a roughened specular signal as well (see [7] Figure ten). Finally, the rough patches might also result from a purely liquid sea surface with wave activity.

The unusually high brightness of all of the areas is sufficiently high so as to only be explicable with a liquid surface: dry, or even merely moist ground will not do. Dry surfaces would also not create the bright-lake effect of reflected sky brightness when seen at high emission angle as shown in Figure 5. Furthermore, the low albedo of Punga Mare could not produce a dry specular reflection of the type seen at Etosha Pan. The brightness constraint then leaves two options for the rough surfaces in Punga Mare: wet mudflats or waves.

If the liquid overlies a solid surface, then it must conform to that surface as a thin layer of liquid. This would be like the bottom part of the specular reflection shown on the sidewalk in Figure 7B. At the central specular point in Figure 7B the water has already drained downhill somewhat (toward the bottom-left in this image), leaving that part of the concrete wet but not presently covered in a layer of water. If the expanses of Punga Mare indicated by the arrows in Figure 2 represent mudflats, they must be almost entirely presently covered in sea liquid (probably a methane/ethane/nitrogen solution [2,28]) because the specular reflection is so bright.

However, in order for that liquid surface to have the measured roughness characteristics the liquid must drape over a solid surface. Such a liquid covering over solid can occur, as evidenced by the liquid-covered sidewalk in Figure 7B. Mudflats on Earth can have very low slopes, but even then it would be difficult to achieve an appropriately thick layer of liquid over an expanse tens of kilometers long. In addition, we see the rough liquid only at discrete locations within Punga Mare. Mudflats might be expected to occur preferentially at the sea’s margin, as at Ontario Lacus [6] (though in detail their distribution would depend on the sea’s bathymetry). The best imaging of Punga Mare to date occurred on T94, as shown at the top left of Figure 4. Unlike the T38 Ontario Lacus data [6], the T94 data do not have the spatial resolution or signal-to-noise ratio needed to discern mudflats. Wetted floating ice [29] would be a similar solution, but would need to be similarly liquid-covered and extensive.

The other possibility is that the bright specular patches represent liquid expanses roughened by wave activity. Wind-induced waves should be possible on Titan [10–12,17], even though searches until now had shown lakes and seas to be perfectly flat [14,15]. The 6° typical slopes within the specular patches is not too dissimilar from typical slopes on Earth’s oceans (4° [30]), particularly given that Hayes et al. [12] predict that Titan waves should be 7 times higher and 2 times steeper than Earth waves produced with the same wind speeds. Indeed, the angular width of sea-surface specular reflections has been used on Earth as a proxy for windspeed for 60 years [31].

**Figure 12:**
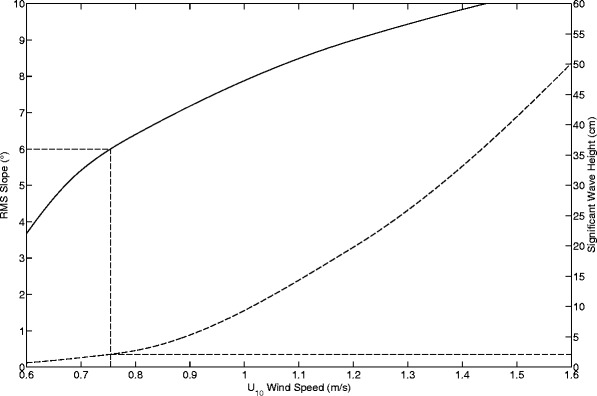
**Slopes, winds, and wave heights.** Here we plot output from the Donelan et al. [32] wave model using the Titan parameters from Hayes et al. [12]. As the solid line we plot the expected RMS surface slope (our *σ*) is as a function of the wind speed as measured 10 meters above the surface (*U*_10_). From that relation we infer winds of 0.76±0.09 m/s in our target areas at the time of the observation. We also plot as the dashed line the significant wave height (defined to be 4 times the RMS in the liquid height across the surface) as a function of *U*_10_, with the scale on the right-hand side of the plot. We therefore infer significant wave heights of $2^{+2}_{-1}$ cm.

We use the equations and parameters from Hayes et al. [12] and derive an explicit wavefield using the model of Donelan et al. [32], adapted to include surface tension effects (i.e., capillary-gravity waves and capillary waves). From these we calculate the expected value for both the wave angle *σ* and the significant wave height (defined as 4 times the root mean square (RMS) surface height) under Titan conditions (Figure 12) and assuming that the liquid viscosity is that of pure methane. Using the slope curve, we infer that our measurement of 6°±1° for surface slopes is commensurate with local winds along our line of sight and at 10 meters altitude of 0.76±0.09 m/s. This inferred windspeed is in broad agreement with the magnitude of winds expected near the threshold for the initiation of wave activity [11,12]. From that wind determination, the expected significant wave heights for this wavefield would be $2^{+2}_{-1}$ cm.

Although not fully replicated by empirical studies, an experiment at 1 bar and Earth’s surface gravity produced 12 mm amplitude waves with winds of 5 m/s [13]. While the wind speed associated with our 6° waves would vary with composition and viscosity, the resulting wavefield is nearly independent of composition. Hence viscosity-related systematic errors afflict the wind speed determination, the significant wave heights are mostly free from compositional systematic errors. Our calculation is in broad agreement with Lorenz et al. [33] who did a similar calculation for RADAR detectivity of waves.

**Figure 13:**
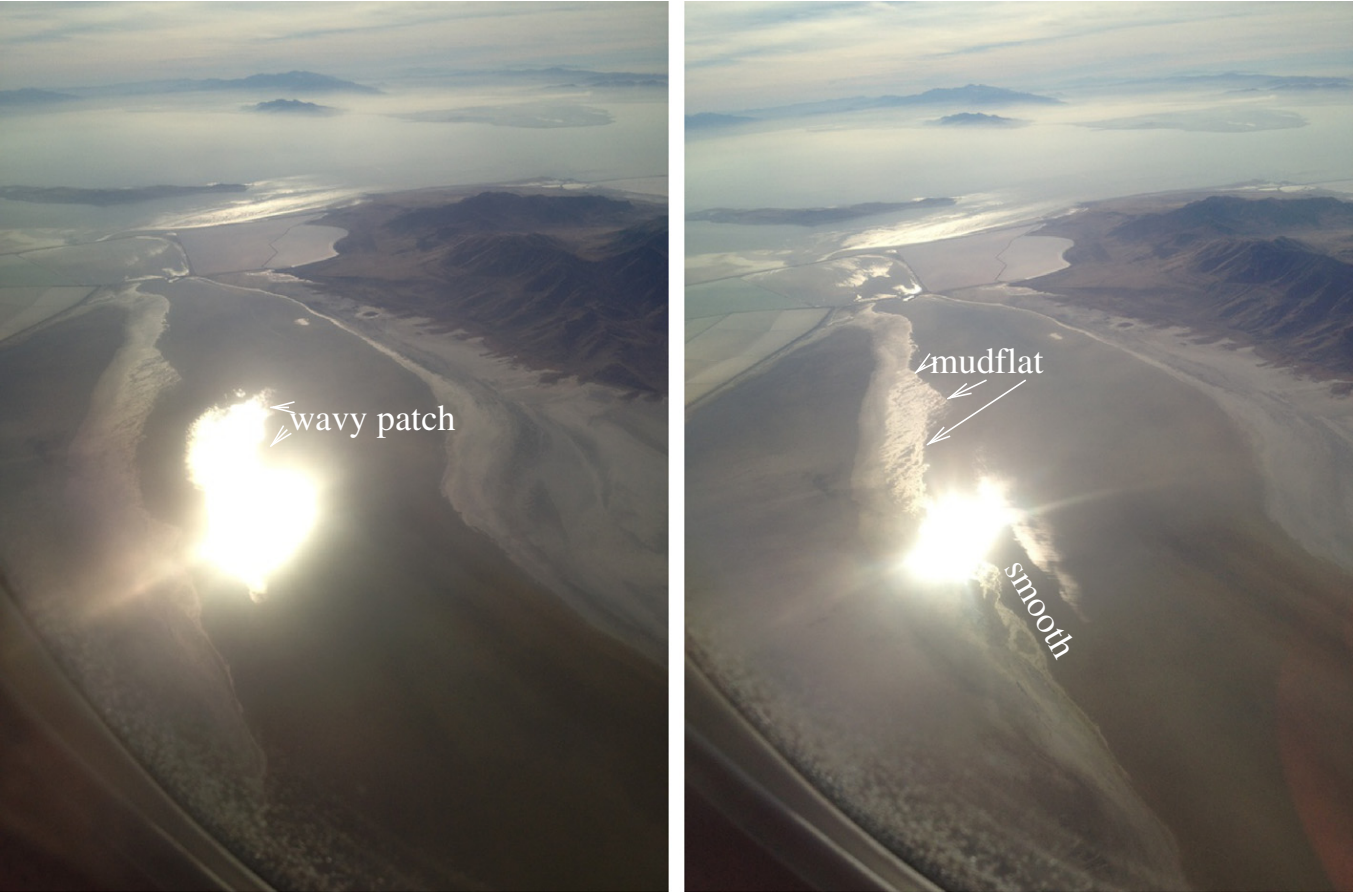
**Great salt lake.** Two photos taken sequentially a few seconds apart looking out the right-hand window while flying southbound on approach to Salt Lake City airport. In the photo at left, the specular point lies within an arm of the Great Salt Lake. Extending above and below the specular point is an area of enhanced wave activity, also showing specular reflection due to its surface roughness. However the area to the left of the specular reflection is wave-free. At right the specular point has moved into that wave-free zone. Now the mudflats to the upper-left of the specular point are starting to show increased specular flux due to their own roughness.

**Figure 14:**
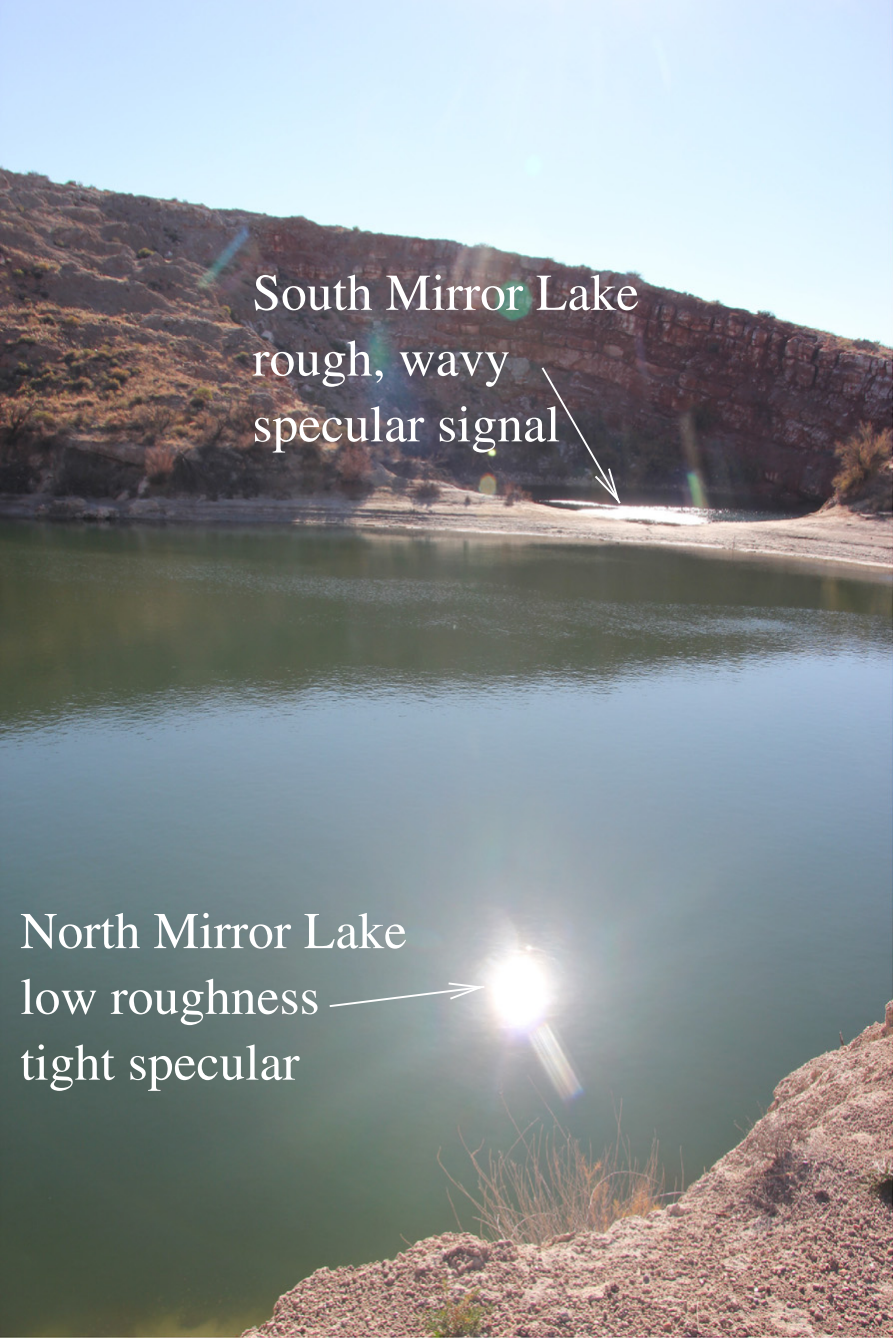
**Bottomless lakes.** Photo of specular reflections off water at Bottomless Lakes State Park, New Mexico, USA. This view shows two lakes — Mirror Lake North at bottom, and Mirror Lake South at top. Mirror Lake North has smaller wave slopes, and thus exhibits a small specular reflection extending around the specular point. However the waves on Mirror Lake South in the distance are high enough so as to show significant specular flux even many degrees away from the specular point. The situation depicted may resemble the situation seen by VIMS on T85, with a relatively clean specular reflection from smooth Kivu Lacus and wave-generated roughness on Punga Mare.

The patchiness of the effect that we see represents a somewhat surprising aspect for waves. Wave activity in one portion of a lake or sea might be expected to propagate throughout the entire expanse, at least to some degree. However Hayes et al. ([12]) show that winds at or just above the threshold for wave generation could be produced locally without such propagation, since the smallest waves are quickly damped by viscous dissipation in the absence of forcing by wind. We show terrestrial analogs in Figures 13 and 14, both cases showing variable sea states (levels of wave activity) both within a single body of liquid (Great Salt Lake in Figure 13) and between adjacent lakes (Bottomless Lakes, New Mexico, USA in Figure 14).

Dissipation occurs more rapidly for short-wavelength capillary waves (waves with surface tension as the restoring force); it is the longer wavelength gravity waves (waves with gravity as their restoring force) that more easily propagate over long distances. Because the first waves that would result from winds just above the threshold velocity would be at the short-wavelength end of where gravity waves are possible, if those waves were incited in patches they would only propagate outside those patches with very low amplitudes.

Thus we posit that our observation may represent waves on Punga Mare that were incited by patchy winds at or just exceeding the wave-generation threshold. If correct, these would represent the first waves on open liquid detected on a body other than the Earth.

Although Global Circulation Models (GCMs) have been used to study the prospects for waves at Ligeia Mare in detail [18], no specific study has done the same for Punga Mare. In general, however, predictions show that in Titan’s arctic the winds should begin to pick up as northern summer approaches [12]. We leave an investigation of whether, and under what conditions, GCMs can replicate this scenario on Punga Mare at the time in Titan’s season of T85 to future work.

If real, the VIMS T85 Punga waves solve the prior paradox of waves’ absence in Titan lakes and seas [17]. Indeed Titan’s maria are liquid and do not have the viscosity of molasses (good for potential lake lander missions [33]). Instead, as suggested by Lorenz et al. [17], wind conditions may not have been favorable for the production of waves until recently.

We note that the patchiness of the putative waves that we see means that it would be possible for observations at a single point or even along a single chord (like [14] and [15]) to miss them. Previously unexplained variations in specular brightness on Kraken Mare on T59 [15] could be due to differing degrees of roughness across the face of the sea.

Had *Cassini* RADAR been observing Punga Mare on T85, could it have seen the putative waves that we describe here? Using the usual Synthetic Aperture RADAR (SAR) mode with high incidence angles (∼30°) it would not detect these waves — in that Bragg regime their signal might be -30 or -40 db [12], well below the single-pixel noise floor of ∼−20 db. At lower incidence angles, however, around 10° or less, the quasi-specular signal should make 6° waves evident even in SAR imaging. *Cassini*’s RADAR has only observed Titan at such low incidence angles once, and in that one observation possible wave activity was observed in one location on Ligeia Mare [34]. Processing *Cassini*’s RADAR data in real aperture mode can beat down the noise and potentially reveal wave signals, but such signals would be convolved with the return from the sea floor [35].

## Conclusion

VIMS T85 observations of Titan’s north pole show specular flux coming from areas within Punga Mare away from the specular point in Kivu Lacus. We develop a numerical model to simulate the appearance of a broad specular reflection off a rough surface on a spherical planet (previous work by [15] assumed very small roughness dispersion and is therefore not appropriate for moderately rough surfaces observed globally). The spectra, locations, and intensities are consistent with a surface covered in liquid and rough at wavelengths much longer than 5 μm with a typical angle of 6°±1°. The inferred surface wind speeds of ∼0.7 m/s are consistent with GCM predictions of increasing wind activity as northern summer approaches (e.g., [36]).

The rough patches could represent either wet mudflats or the development of waves on the sea surface. Because such mudflats would need a thin layer of liquid draped over rough mud to be consistent over areas tens of kilometers across, we prefer the waves interpretation. Future observations could definitively differentiate between the two ideas: if the regions are consistently rough as a function of time, then they are likely mudflats, whereas if the rough areas are different on a future flyby then that observation would be more consistent with waves.

The patchy nature of the putatively wavy seas implies locally variable winds near the threshold for wave generation. While future specular observations of the quality seen on T85 will be rare, there will be a few. In particular, a specular observation is planned for T101 on 2014 May 17. Combining the T85 observation with those future measurements should allow us to piece together a time-resolved picture of the frequency and intensity of high-wind events across Titan’s north polar seas. The *Cassini* RADAR instrument also has prospects for detection of wave activity in the future through low-incidence synthetic aperture radar observations, bistatic experiments (T101, T102, and T106) and an altimetry pass over Kraken Mare (T104). Observations from *Cassini* or other imaging missions such as JET (Journey to Enceladus and Titan) [37] or an airplane [38] or balloon could monitor wave activity in the future by planning observations of the specular point at high phase at close enough range for the roughness effect to be seen.
